# Strategies for the implementation of best practice guidelines in operating theatres: An integrative literature review

**DOI:** 10.4102/hsag.v26i0.1488

**Published:** 2021-06-29

**Authors:** Olukemi O. Owolabi, Portia J. Jordan, Margaret Williams, Wilma ten Ham-Baloyi

**Affiliations:** 1 Department of Nursing, Faculty of Health Sciences, Nelson Mandela University, Port Elizabeth, South Africa; 2 Department of Nursing and Midwifery, Faculty of Medicine and Health Sciences, Stellenbosch University, Cape Town, South Africa; 3 Department of Nursing, Faculty of Community and Health Sciences, University of the Western Cape, Cape Town, South Africa; 4 Faculty of Health Sciences, Nelson Mandela University, Port Elizabeth, South Africa

**Keywords:** operating theatre, nurse, implementation, strategies, best practice guidelines, evidence, integrated literature review, practice

## Abstract

Best practice guidelines (BPGs) exist for operating theatre (OT), but strategies to implement them are lacking. To address the gap, an integrative review was undertaken to identify strategies which can be used to implement BPGs in OT. This article aimed to summarise the best existing literature in order to identify and describe strategies for the implementation of BPGs in OT. An extensive search was undertaken to include relevant literature from February 2005 to March 2020 using the following databases: CINAHL, Medline, Biomed Central, Academic Search Complete and Health Source: Nursing/Academic Edition (EBSCOhost) and the Cochrane library. This integrative literature review followed the methodology proposed by Whittemore and Knafl, namely: (1) identification of the research problem, (2) search of the literature, (3) evaluation of the data, (4) analysis of the data and (5) presentation of the results. On completion of the critical appraisal, 15 (*n* = 15) articles met the inclusion criteria and relevant data were synthesised. The review identified six strategies facilitating implementation of BPGs in OT, namely, communication, education materials and mass media, academic detailing, opinion leaders, audit and feedback, and teamwork and collaboration. The review validated strategies for the implementation of BPGs in OT. Implementation of BPGs is essential to both provide and improve patient care and to benefit health outcomes. This review is expected to contribute to the provision of strategies to implement BPGs in OT.

## Introduction

The provision, by healthcare providers, of a safe environment for patients undergoing surgical procedures is crucial. The operating theatre (OT) is a unique unit in which complex clinical care is provided by highly trained interdisciplinary teams, using high-cost procedures and a large array of supplies, instruments and surgical implants that can be difficult to manage during surgical procedures (European Operating Room Nurses Association [EORNA] 2019:3). Operating theatre nurses are trained to be aware of risks related to patient mismanagement, including those related to musculoskeletal injuries, patient misidentification, surgical site infection and equipment.

To reduce the risks of patient mismanagement, it is recommended that clinical practices be based on the best available evidence in the form of best practice guidelines (BPGs). The implementation of BPGs provides best available evidence to support clinical decision-making to improve quality care, good patient outcomes and cost effectiveness (Melnyk et al. 2017:6). Well-written BPGs should be used to optimise healthcare delivery and improve patient outcomes (Ayabe et al. 2017:22).

The Association of peri-Operative Registered Nurses (AORNs) provide guidance related to the safety of peri-operative patients and healthcare personnel, with the aim of establishing best practice and implementing safety measures in all peri-operative practice settings (AORN 2019:710). Despite the wide spectrum of BPGs for use in OT, implementation remains inadequate (Vogelsang et al. 2019:5). Strategies are needed to assist or facilitate the implementation and use of BPGs in the OT (Vogelsang et al. 2019:5).

Finding the most appropriate strategy to successfully implement BPGs in OTs is imperative in order to improve OT safety. This review aims, therefore, to summarise the best existing literature in order to identify and describe strategies for the implementation of BPGs in OT.

## Methods

The review was conducted in accordance with Whittemore and Knafl’s (2005:547) methodology for integrative literature reviews, including five stages, namely: (1) identification of the research problem, (2) search of the literature, (3) evaluation of the data, (4) analysis of the data and (5) presentation of the results. The review was conducted by the first author, under the supervision of the other three authors who evaluated the reliability of each strategy selected for inclusion in the review. This article presents the findings of the integrative review section of a larger study aimed at developing strategies to facilitate the implementation of BPGs in OT (cf. Owolabi 2020).

### Stage one: Identification of the research problem

After the identification of the research problem that although finding the most appropriate strategies to assist or facilitate the implementation and use of BPGs in the OT is needed, no integrative literature review has been conducted to summarise the best literature on existing strategies in this context, the following question was used for the integrative literature review: ‘What literature is existing regarding strategies for the implementation of BPGs in OT?’

### Stage two: Search of the literature

The search strategy aimed to identify all eligible human studies pertaining to the integrative review question and included both published and unpublished literature. Databases searched included The Cumulative Index to Nursing and Allied Health Literature (CINAHL), Medline, Biomed Central, Academic Search Complete, Health Source: Nursing/Academic Edition (EBSCOhost) and the Cochrane library. A manual search was performed using websites of the following organisations: the Association of peri-Operating Room Nurses (AORN), Centres for Disease Control (CDC), US National Guideline Clearinghouse, the Guidelines International Network (G-I-N), the National Institute for Health and Clinical Excellence (NICE), Scottish Intercollegiate Guidelines Network, Royal College of Nurses and the Registered Nurses Association of Ontario (RNAO). In addition, grey literature was searched to include published and unpublished theses and dissertations.

The following search terms were used: ‘implementation’, ‘strategy’ and further search terms that indicate aspects of ‘compliance, acceptance, adherence’ as indicated in a strategy* (Title/Abstract) OR guidance* (Title/Abstract) OR clinical practice* best practice* (Title/Abstract) AND (strateg*[Title/Abstract] OR barrier* [Title/Abstract]) AND implement* (Title/Abstract) AND OR accept* (Title/Abstract) OR approv* (Title/Abstract) OR adopt* evidence based* OR operating theatre*.

#### Inclusion criteria

Inclusion criteria for the integrative review included literature from all levels of evidence (adapted from Melnyk & Fineout-Overholt 2011), published in English to avoid translation costs, from 2005 to 2020, related to the implementation of BPGs in the OT. As a preliminary search found a dearth of literature in the OT context, literature related to the implementation of BPGs in similar, demanding contexts (such as acute care, high acuity care or Intensive Care Unit (ICU)) and BPGs for the implementation of best practices in a general healthcare context (including the OT context) were included.

#### Exclusion criteria

All literature that did not pertain to nurses (either as individual professionals or as part of the interprofessional team) or did not focus on best practice guideline strategies for healthcare were excluded.

### Stage three: Evaluation of the data

The evaluation process, which included screening, selection and critical appraisal of articles, was conducted by two reviewers (O.O. and W.T.H.B.) independently. Discrepancies were assessed and disagreements were resolved via discussion between the two reviewers. All identified sources relevant to the integrative review question were considered. Titles and abstracts were screened independently for adherence to the stipulated inclusion criteria by the two reviewers. A similar process was used for the full text inclusion based on the specified inclusion criteria.

**FIGURE 1:**
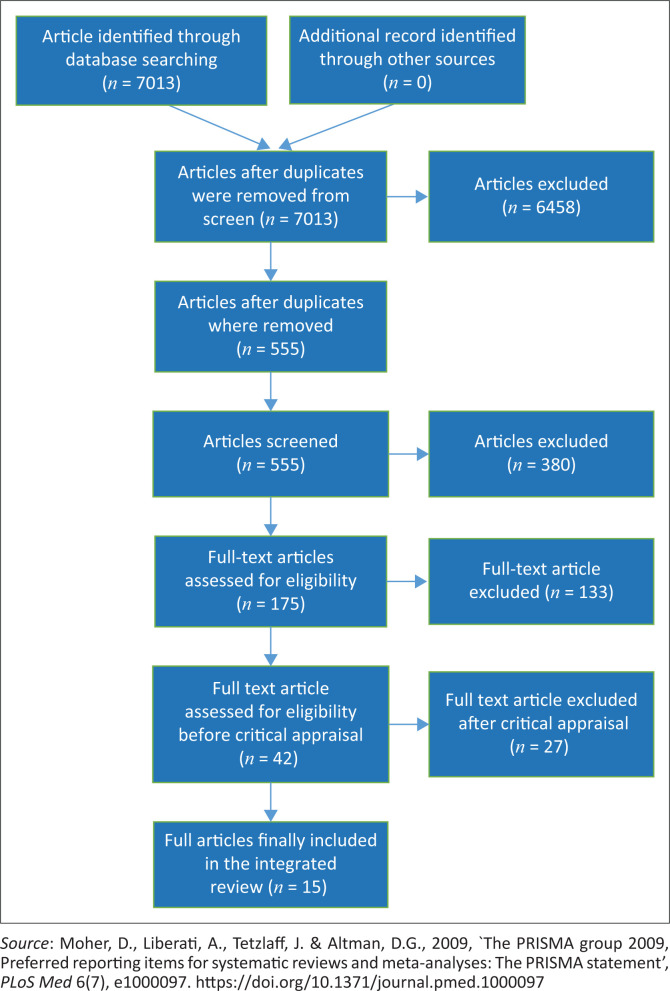
Preferred reporting items for systematic reviews and meta-analysis diagram of literature search.

The procedures used for and the results of the search are depicted in the Preferred Reporting Items for Systematic Reviews and Meta-Analysis (PRISMA) flow chart (Figure 1). Of the 7013 articles that were screened, 6458 articles were excluded as they either did not fit the inclusion criteria or they were duplicates (*n* = 380). After further removal of duplicates and screening, a total of 175 full-text articles were screened for a third time of which 42 articles were considered for critical appraisal. Out of the 42 relevant articles that were assessed for methodological quality, 27 articles were excluded, leaving 15 articles for analysis.

The selected studies were critically appraised independently for methodological quality by the two reviewers using various critical appraisal tools (Table 1).

**TABLE 1:** Critical appraisal tools used.

Type of review design	Critical appraisal tool used
Randomised controlled trials	Joanna Briggs Institute (JBI) appraisal tools for randomised controlled trials – RCTs (JBI)
Best practice guidelines	Appraisal of guidelines for research and evaluation instrument (AGREE II)
Systematic reviews	Systematic reviews (JBI)
Quantitative pre-test, post-test studies	Long et al. (2002), appraisal tools
Qualitative studies	Joanna Briggs Institute qualitative appraisal tools
Mixed methods	Johns Hopkins evidence appraisal instrument and appraisal tools
Non-research narrative reviews (opinion experts and reports of or expert committees/conference papers)	Johns Hopkins non-research tools

To include the most rigorous evidence, an appraisal score of 70% and above was considered the cut-off score (depending on the appraisal tool used). For critical appraisal tools with items requiring a ‘yes’ or ‘no’ answer, ‘yes’ scored 1. The total score and a percentage were calculated. For the appraisal of guidelines for research and evaluation (AGREE II) instrument, every item in the domain had a minimum score of 1 and a maximum of 7, totalling 161 for all items under the 6 domains (Brouwers et al. 2013:16). The final selection of articles for inclusion in the integrative review based on the appraisal score was completed after the consensus was reached by the two reviewers.

After critical appraisal, the data from the included studies were manually extracted by two independent reviewers. The extracted data included details about the context, type of study, methods and key findings relevant to the review question. Disagreements were resolved through discussion, and there was no need to consult a third reviewer. A total of *n* = 27 articles were excluded because of poor rigour.

### Stage four: Analysis of the data

As a result of the heterogeneity of the included studies, a meta-analysis or meta-synthesis could not be conducted. Therefore, extracted findings of the 15 articles were synthesised using a thematic analysis approach based on the major recommendations for the development of strategies for the implementation of BPGs relevant for the OT.

### Stage five: Presentation of the results

Interpretation of the extracted data in relation to the review question is the final phase of the integrative literature review. The results were presented in narrative summary form, supported by a figure, where applicable.

### Ethical considerations

Ethical clearance was obtained from the Faculty of Postgraduate Studies Committee (FPGSC) of Nelson Mandela University, reference number: H16-HEA-NUR-009. Plagiarism was avoided by giving credit to all authors of the applicable literature.

## Review findings

A total of 15 (*N* = 15) articles were included in the review (see Online Appendix 1). These included three systematic studies, one randomised controlled trial (RCT), three mixed methods RCTs, three qualitative reviews and five non-research documents (see Table 2).

**TABLE 2:** Types of evidence included in the review and per the hierachy of evidence. (*N* = 15).

Level of evidence	Type of evidence	Number of studies
I	Systematic review studies	3
II	Randomised controlled trial and mixed method randomised controlled trial	4
IV	Qualitative review	3
VII	Non-research documents (opinion experts and reports of or expert committees/conference papers)	5

The total number of articles directly targeting the OT context were only five, 33% of all included articles (Emond et al. 2015; Gotlib et al. 2015; Häggman-Laitila, Mattila & Melender 2016; Landers 2015; Munten et al. 2010), whilst one was from the acute hospital care context (Breimaier, Halfens & Lohrmann 2015), two from the high acuity context (Chan et al. 2017; Harris et al. 2015), two from the ICU context (Ploeg et al. 2016; Wallen et al. 2010) and five that were conducted in a general healthcare/clinical care context (Friedman et al. 2009; Guerrerro et al. 2016; Holleman et al. 2009; Newhouse et al. 2012; Shatpattananunt, Petpichetchian & Kitrungrote 2015).

### Thematic presentation of data

**FIGURE 2:**
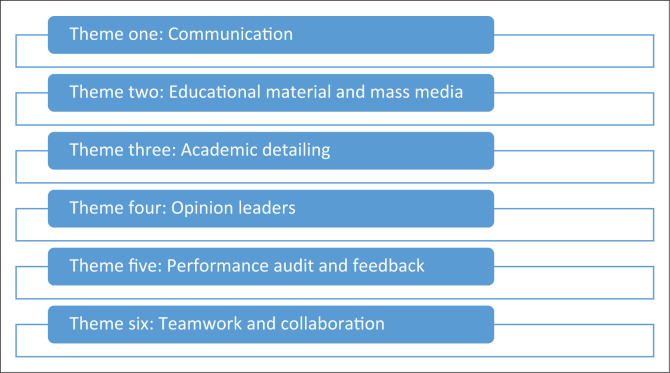
Six themes identified from analysis of data.

Six themes were derived from the data extracted from the 15 articles (see Figure 2).

#### Theme 1: Communication

Data extracted from 9 out of the 15 articles concurred that optimal and diverse communication methods enhance and contribute to the implementation of BPGs (Chan et al. 2017; Emond et al. 2015; Friedman et al. 2009; Gotlib et al. 2015; Holleman et al. 2009; Landers 2015; Shatpattananunt et al. 2015; Wallen et al. 2010).

The relevance of communication in the implementation of best practice was supported by literature. Gotlib et al. (2015:3) discussed the necessity of using different communication methods and forms of communication channels. Face-to-face communication should be emphasised, especially to resolve conflicts or crises in practice (Gotlib et al. 2015:3).

Wallen et al.’s (2010:2761) study on multifaceted mentorship programmes designed to implement BPGs in a clinical research-intensive environment suggested that channels through which BPGs are communicated to healthcare professionals can shape the ways they engage with, and use, information such as clinical aides and online training programmes. Emond et al. (2015:9) added that information should be communicated via credible sources such as government departments, professional bodies and peers.

Furthermore, there are different communication channels through which OT nurses learn about BPGs, such as online training programs, posters and workshops. Chan et al. (2017:4) reviewed 15 studies, using interrupted time-series designs, to demonstrate that mass media, for example, television and radio, targets populations such as providers and patients and can affect the implementation of BPGs.

Friedman et al. (2009:223) provided important insights into the design of action plans to integrate supportive BPGs into day-to-day practice. Monitoring interventions were used to encourage adherence to BPGs amongst participating staff. This was carried out by scheduling staff to permit participation in the interventions, providing staff with reminders to complete web-based exercises and using emails as a source of communication. Holleman et al. (2009:1258) believed that communication amongst OT nurses, including listening, supports constructive conflict resolution by agreement on procedures for decision-making and mutually negotiated work boundaries. Teams should openly share information about the implementation of BPGs in OT units through their group meetings.

In summary, effective communication on the implementation of BPGs can enhance stronger relationships between OT nurses and managers. This would assist in the implementation of BPGs and enhancing relationships with other members of the OT unit.

#### Theme 2: Educational materials and mass media

Of the 15 articles appraised, four highlighted education and the use of mass media (local or national radio, magazines, the Internet and terrestrial, cable and satellite television) as being effective methods to promote and/or facilitate the implementation of BPGs. Shatpattananunt et al. (2015:365) recommended guidelines, posters, audiovisual materials and electronic publications to improve clinical care. Breimaier et al. (2015:1744) concurred that implementation of BPGs on fall-prevention benefited from the use of printed educational materials. Gotlib et al. (2015:11) highlighted the dissemination of complete guideline documents and abridged summaries as a reliable and strategic approach to inform staff about BPGs. Munten et al. (2010:136) reported that promoting the uptake of research evidence in clinical practice using mass media is advisable. Shatpattananunt et al. (2015:368) indicated that educational meetings and materials result in moderate improvements in the implementation of BPGs and yield better outcomes in patient management and care.

Our results suggest that the use of educational materials and mass media will have a significant effect on augmenting BPGs implementation in the OT. More specifically, this will increase awareness of BPGs, thereby encouraging behavioural change and promoting acceptance of BPGs to improve patient care in OT.

#### Theme 3: Academic detailing

Five articles highlighted the importance of academic detailing for BPGs implementation (Chan et al. 2017:5; Guerrero et al. 2016:9; Harris et al. 2015:11; Holleman et al. 2009:55). Academic detailing, or educational outreach, as applied to BPGs, involves interactive face-to-face education of individual practitioners in their practice setting by an educator (usually a clinical facilitator or nurse educator) with expertise in a particular topic (such as hand washing or surgical scrubbing). This is one approach to changing practice to better align with the provision of BPGs. The goal of academic detailing is to improve nurses’ decision-making through unbiased information, enhance BPGs in patient care and improve patient outcomes (Chan et al. 2017: 6).

Academic detailers are able to explain the research basis for the BPGs and to respond convincingly to challenges and debates (Harris et al. 2015:11). They can deliver feedback about a provider or about team performance regarding selected BPGs (e.g. in respect of frequency of pain assessment) (Holleman et al. 2009:55). Harris et al. (2015:11), reporting on a best practice programme for the introduction of new technologies and clinical practices, revealed that an academic detailer in the context of OT can be anyone in the unit who understands and can explain the research aspects of BPGs to their colleagues.

Guerrero et al. (2016:9) indicated that academic detailing promotes positive changes in the practice behaviour of clinical practitioners. Chan et al. (2017:5) stated that academic detailing has a larger impact than audit and feedback. Finally, Ploeg et al. (2016:212) confirmed that evaluating the effectiveness of academic detailing should include measuring changes in OT nurses’ decision-making and be centred on good patient outcomes.

In this section, evidence from the literature establishes that when academic detailing is conducted amongst OT nurses, implementation of BPGs will be encouraged, which should result in the improvement of the quality of care and patient outcomes. The implementation of academic detailing will assist OT nurses to positively change their attitudes towards the implementation of BPGs in OTs by encouraging improvement in decision-making during patient care in areas of knowledge and attitude in their daily practice.

#### Theme 4: Opinion leaders

Altogether 8 of the 15 articles (Gotlib et al. 2015:29; Guerrero et al. 2016:19; Harris et al. 2015:5) indicated that an opinion leader is an individual from the local peer group of nurses in the OT who is viewed as a respected expert in the implementation of BPGs. This person is considered by colleagues to be technically competent and is trusted to make appropriate connections between the evidence base of practice and the local situation. Nursing managers, for example, may fulfil this role. Opinion leaders’ roles and functions are considered important during the implementation of BPGs and are affected by the degree to which those individuals are able to influence other individuals’ attitudes in a desired way.

The use of opinion leaders in OTs improves the ability of OT nurses to implement BPGs in the care of a patient (Holleman et al. 2009:1262). Newhouse et al. (2012:83) explained that an opinion leader’s support is important for promoting the implementation of BPGs. This support would ensure provision of necessary resources, materials and time in order to fulfil assigned responsibilities. According to Ploeg et al. (2016:214), opinion leaders in health systems need to create an organisational mission and strategic plan and to implement staff performance expectations to incorporate the use of BPGs’ recommendations.

Robust leadership is critical to encourage organisational growth, such as reduction in convergent thinking and routines amongst OT nurses and incorporation of BPGs in their practice to improve patient outcomes (Chan et al. 2017:5). An organisation that is able to systematically identify, capture, interpret, share and reframe new knowledge and to use it appropriately will be better placed to assimilate BPGs (Shatpattananunt et al. 2015:363). Opinion leaders’ enhanced capacity for objective assessment, combined with insight and a positive attitude in the face of difficulty, will achieve the best outcomes. There should be mutual respect, cooperation and effective communication between OT nurses and opinion leaders (Shatpattananunt et al. 2015:363).

The literature reviewed establishes that it is important to identify opinion leaders who are able to influence OT nurses in the unit to implement BPGs through teaching and advocating for the intended change in current practice.

#### Theme 5: Performance audit and feedback

A total of 7 out of 15 articles (Breimaier et al. 2015:1744; Chan et al. 2017:5; Emond et al. 2015:2; Gotlib et al. 2015:345; Holleman et al. 2009; Shatpattananunt et al. 2015:363; Wallen et al. 2010:2763) recommended audit and feedback as a continuous process of measuring performance (of both process and outcome), aggregating data into reports and discussing findings with OT nurses. Audit and feedback are defined as the summary of clinical performance of healthcare at a specified period of time in order to change the behaviour of professional healthcare workers regarding a specific practice (Gotlib et al. 2015:345).

Such ‘auditing’ involves collecting data or information at the individual clinician or practice level. The ‘feedback’ portion of the audit and feedback generally involves the use of reports that are provided to individual clinicians (Gotlib et al. 2015:345). Emond et al. (2015:9), Friedman et al. (2009:226) and Gotlib et al. (2015:345) observed that hard copy or electronic health records are used for audit and feedback as these records are expected to reflect the assessments, interventions and outcomes associated with the delivery of care.

Audit and feedback include recommendations for action used to increase group awareness of their own or others’ practice (Chan et al. 2017:5). Audit and feedback help OT nurses to monitor improved care processes and patient outcomes (Wallen et al. 2010:2763).

Audit and feedback assist OT nurses in establishing where they are falling short in the use of BPGs, allowing implementation of improvements, re-audit or closing of the audit cycle and gauging if change has taken place (Chan et al. 2017:11). Five studies, namely, Emond et al. (2015), Chan et al. (2017:5), Breimaier et al. (2015:1745), Holleman et al. (2009), Shatpattananunt et al. (2015:363), highlighted the following aspects of using data feedback in the management of surgical patients:

- Feedback data must be perceived by nurses as important and valid.
- Data sources and timeliness of data feedback are critical to perceived validity.
- Establishment of credibility of data within a hospital takes time.
- Validity of data feedback is improved by benchmarking.
- Effectiveness of data feedback can be enhanced by nursing leaders.

Accordingly, data feedback provides nurses with organised and automatic information on the quality of care delivered to patients in OT following the implementation of BPGs.

#### Theme 6: Teamwork and collaboration

A total of 3 of the 15 articles describe teamwork and collaboration as critical factors for the successful implementation of guideline (Emond et al. 2015:6; Holleman et al. 2009:1258; Landers 2015:662). Teamwork is identified as key in the implementation of BPGs within OTs, with members who are highly skilled and motivated helping to launch change initiatives. For example, OT nurses can greatly influence the success of using a safe surgery checklist because they are active participants in surgical procedures and often initiate the process.

Collaboration involves interdisciplinary members working together in consultation, ensuring utilisation of various cadres of nurses during patient care (Emond et al. 2015:6; Landers 2015:662). Collaboration is the ability to function effectively within nursing and inter-professional teams by fostering open communication, mutual respect and shared decision-making to achieve quality patient care (Emond et al. 2015:6). Staff members are more likely to identify collaboration within the practice team as an important instrument for strategy implementation (Landers 2015:662).

In general, teamwork will provide OT nurses with the knowledge, skills and abilities needed to work collaboratively within the OT and to communicate more effectively about BPGs implementation with other members of the team, such as the surgeon and anaesthetist. An inter-professional teams’ collaboration approach has the potential to improve the functioning of a team during the implementation of BPGs.

In summary, the results of the review show that collecting data or information at the individual level, or practice in the form of teamwork and collaboration will ensure that OT nurses can see how their efforts improve patient care and outcomes during the period of data collection. This will also allow nurses to reflect on the progress made during the implementation process. This can only be achieved by discussion of the facts concerning BPGs rather than OT nurses being passive recipients of feedback reports.

## Discussion

This review aimed at exploring and describing the best available literature regarding strategies for the implementation of BPGs in OTs. Six major themes emerged from our analysis of strategies, namely, ‘communication, educational materials and mass media, academic detailing, opinion leaders, performance audit and feedback and teamwork and collaboration’. Effective communication on BPGs implementation can enhance relationships between OT nurses and their managers to achieve identified goals and enhance interdisciplinary relationships with the members of the OT unit.

Our review findings were in accordance with the findings of Barreto (2018:1), who demonstrated that the development of strategies for the implementation of BPGs is the key step required to move OTs into the next level of practice. The best practice implementation was found to be highly dependent on the local context, the people involved and available resources (Barreto 2018:1). Therefore, management should be involved in implementation planning and execution of plans pertinent to their individual institutions.

In the context of OT, quality standards attained in various health systems will depend on the dissemination of the best evidence available during management of the patient (Blomberg, Bisholt & Lindwall 2018:420). Association of peri-Operative Registered Nurse (AORN 2017:2) develops evidence-based standards of responsibility and safety for patient care. The use of these standards will show the OT nurse where to exercise judgement, based on education and experience, to determine appropriate care for the patient.

This review establishes that the role of strategies for the implementation of BPGs is to provide clear and well-informed recommendations to be used as support for OT nurses when making decisions during the nursing care of patients. Tucker and Gallagher-Ford (2019:50) indicated that the implementation of BPGs is a complex and resource-intensive step. Significantly, the time and effort needed is regularly underestimated and under-resourced. Tucker and Gallagher-Ford (2019:50) reported selecting strategies aligned with clinical practice to address organisational culture and leadership structure and support when implementing BPGs.

Jowsey et al. (2019:1) implemented a multidisciplinary teamwork strategy amongst OT nurses in New Zealand public hospitals over 5 years to identify, at an early stage of implementation, which strategies work and which require modification.

The RNAO (2017:49) provided a summary of strategies for the implementation of BPGs during perioperative management of patients. The strategies describe the invaluable contribution of nurses who have studied informatics and applied relevant competencies during the implementation of BPGs. In the RNAO’s (2017:49) study, nurses ‘combined their clinical knowledge with an understanding of the information requirements of nurses and the use of technology in the nursing environment’ to positively influence the design of the system and increase implementation.

Opinion leaders should influence OT nurses through teaching and advocating for change in current practice, to ensure and enhance the implementation of BPGs. Our results are supported by Cranley et al. (2017:2) who identified nine strategies used in the implementation of BPGs: opinion leaders, coaches, champions, research facilitators, clinical/practice facilitators, outreach facilitators, linking agents, knowledge brokers and external–internal facilitators. These nine strategic role players provide definition, key features, training requirements and key personal attributes and skills for the effective implementation of BPGs (Cranley et al. 2017:2).

This review strengthens results from the existing literature used for this review by stating that the findings from the six strategies discussed here ought to assist in the implementation of BPGs in OT.

### Implications

The outcome of this review is expected to contribute to policy by serving as a basis for re-strategising OT nursing practice through the provision of clear policy direction on and strategies for the implementation of BPGs in OT.

### Recommendations

To implement BPGs into OT nursing practice, several strategies will be needed. It will be necessary to remain aware that what works in one context of care might not work in another setting. Accordingly, a context analysis should be conducted in order to determine the best strategies to facilitate the successful implementation of BPGs in the selected OT.

- Nursing practice:
  ▪ The study developed the strategies for the implementation of BPGs for the OT.
- Nursing research:
  ▪ Operation theatre nurses should be encouraged to conduct research to update the strategies so as to increase their knowledge, attitude and practice on BPGs, as well as to maintain high standards of care for surgical patients.
  ▪ Operation theatre nurses should be motivated to search for and use BPGs available in their different units, in order to get a clearer vision of what each procedure entails and the best practices that underpin each surgical procedure.
- Nursing education:
  ▪ Educational presentation to OT nurses should be based on BPGs that have been peer reviewed and which include a variety of teaching and learning strategies.
  ▪ Information sessions should be presented to the OT sectors to make the findings of the developed strategies available to those theatres, where the preparation and support needs for the implementation of BPGs is necessary.

### Limitations

There are few studies of implementation of BPGs that relate to low middle-income countries. Most studies accessed were from middle to high-income countries, making it difficult to compare results. The literature search was as thorough as possible but was limited to the databases that could be accessed via the university’s subscription.

## Conclusion

The aim of the integrative literature review was to summarise the best existing literature in order to identify and describe strategies for the implementation of BPGs in OT. The results of the review will facilitate the implementation of BPGs in OTs in South Africa. In the light of the available evidence, we believe that the six strategies discussed in this review could bring about improvements in patient outcomes and the health of patients and further improve the practice of OT nurses in order to achieve positive patient outcomes.
